# Prognostic impact of R1 resection margin in synchronous and simultaneous colorectal liver metastasis resection: a retrospective cohort study

**DOI:** 10.1186/s12957-023-03042-5

**Published:** 2023-06-07

**Authors:** Na Reum Kim, Essam Dhafer Alhothaifi, Dai Hoon Han, Jin Sub Choi, Gi Hong Choi

**Affiliations:** 1 Department of Surgery, Division of Hepato-Biliary and Pancreatic Surgery, Yonsei University College of Medicine, Seoul, Republic of Korea; 2 Department of Surgery, Division of Hepato-Biliary Pancreatic Surgery, King Saud Medical City, Riyadh, Saudi Arabia

**Keywords:** Colorectal cancer liver metastasis, R1 resection, Long-term outcome, Chemotherapy, Prognostic factor

## Abstract

**Background:**

A margin ≥ 1 mm is considered a standard resection margin for colorectal liver metastasis (CRLM). However, microscopic incomplete resection (R1) is not rare since aggressive surgical resection has been attempted in multiple and bilobar CRLM. This study aimed to investigate the prognostic impact of resection margins and perioperative chemotherapy in patients with CRLM.

**Methods:**

A total of 368 of 371 patients who underwent simultaneous colorectal and liver resection for synchronous CRLM between 2006 and June 2017, excluding three R2 resections, were included in this study. R1 resection was defined as either abutting tumor on the resection line or involved margin in the pathological report. The patients were divided into R0 (*n* = 304) and R1 (*n* = 64) groups. The clinicopathological characteristics, overall survival, and intrahepatic recurrence-free survival were compared between the two groups using propensity score matching.

**Results:**

The R1 group had more patients with ≥ 4 liver lesions (27.3 vs. 50.0%, *P* < 0.001), higher mean tumor burden score (4.4 vs. 5.8%, *P* = 0.003), and more bilobar disease (38.8 vs. 67.2%, *P* < 0.001) than the R0 group. Both R0 and R1 groups showed similar long-term outcomes in the total cohort (OS, *P* = 0.149; RFS, *P* = 0.414) and after matching (OS, *P* = 0.097, RFS: *P* = 0.924). However, the marginal recurrence rate was higher in the R1 group than in the R0 group (26.6 vs. 16.1%, *P* = 0.048). Furthermore, the resection margin did not have a significant impact on OS and RFS, regardless of preoperative chemotherapy. Poorly differentiated, N-positive stage colorectal cancer, liver lesion number ≥ 4, and size ≥ 5 cm were poor prognostic factors, and adjuvant chemotherapy had a positive impact on survival.

**Conclusions:**

The R1 group was associated with aggressive tumor characteristics; however, no effect on the OS and intrahepatic RFS with or without preoperative chemotherapy was observed in this study. Tumor biological characteristics, rather than resection margin status, determine long-term prognosis. Therefore, aggressive surgical resection should be considered in patients with CRLM expected to undergo R1 resection in this multidisciplinary approach era.

**Supplementary Information:**

The online version contains supplementary material available at 10.1186/s12957-023-03042-5.

## Background

Colorectal cancer (CRC) is one of the most common cancers worldwide. Liver metastases occur more frequently in CRC. Approximately 25% of patients with CRC were diagnosed with liver metastases on initial evaluation [1–6]. Hepatic resection is considered the only curative therapy for colorectal liver metastasis (CRLM); however, 20–30% of these lesions are resectable at the time of diagnosis.

In the past, the inability to achieve a 1-cm clear margin was generally considered an absolute or relative contraindication to surgery owing to higher disease recurrence and poor OS [7, 8]. Improvements in the surgical technique, optimization of perioperative care, developments in diagnostic imaging, and effectiveness of chemotherapy regimens have expanded the resection criteria for CRLMs. Currently, more than 1-mm cancer-free margin is considered the standard resection margin for CRLM [9–13]. Furthermore, the resection margin status was considered less important in the past, and liver resection was aggressively performed for patients with multiple or bilateral CRLM [14, 15]. Moreover, when the CRLM is in contact with vascular structures, detachment from the vessels is feasible owing to improvements in the liver resection techniques based on intraoperative ultrasound [16–18] which resulted in comparable postoperative surgical outcomes for R0 resection [19]. Several studies have reported 5-year overall survival (OS) rates of up to 58% [20–23]. Recently, as parenchymal sparing hepatectomy (PSH) has been technically performed safely in CRLM patients, bilobar multiple CRLMs can be removed with enough remnant liver volume [24]. Additionally, PSH is more likely to undergo repeated hepatectomy, which is the optimal treatment for recurrent liver lesions, resulting in an improvement of up to 72.4% in 5-year OS in patients who occurred liver recurrence after PSH [25].

Even in experienced institutions, the reported R1 resection rate was not small (10–30% of patients); moreover, in advanced CRLM, the rate was higher [12, 14, 15, 26–28]. With the application of preoperative chemotherapy, resection was increasingly available even in initially unresectable diseases. Some studies showed significantly inferior disease-free survival in R1 resection without preoperative chemotherapy compared to R0 resection; however, it was not considered a negative prognostic factor in patients who received preoperative chemotherapy [11, 12, 29]. Our study aimed to compare the clinicopathologic characteristics and oncologic outcomes between R0 and R1 resection in patients who underwent simultaneous resection for synchronous CRLM. The prognostic impact of resection margins was further analyzed according to whether patients received preoperative chemotherapy.

## Methods

### Patient selection

This retrospective cohort study was approved by the institutional review board of our institution, which waived the requirement for informed consent. Between 2006 and June 2017, 371 consecutive patients underwent simultaneous colorectal and liver resection for synchronous CRLM at the Severance Hospital (No. 4–2022-0696). Three patients who underwent R2 resection were excluded from the study. The patients were divided into two groups according to the liver resection margin status: R0 and R1. R0 resection included no tumor in the surgical margin. R1 resection included either abutting tumor on the resection line or involved margin in the pathological report. The nearest margin was used in patients who underwent multiple liver resections. Additionally, R1 resection was classified as vascular R1 resection (Vas R1, the intraoperative detachment of CRLM from a major intrahepatic vessel) and parenchymal R1 resection (Par R1, when CRLM are exposed along the transection plane), and subgroup analysis was performed between Vas R1 and Par R1 resection.

For each patient, the demographics and clinicopathological characteristics of primary CRC, such as initial carcinoembryonic antigen (CEA), the 8th American Joint Committee on Cancer T stage and N stage, location (right-sided colon vs. left-sided colon vs. rectum), and differentiation grade were recorded. All the patients underwent preoperative screening to assess the extent of liver metastases using clinical examination and imaging studies, computed tomography (CT), and magnetic resonance imaging. The pathologic data of liver metastases included the number of lesions, largest tumor size, bilobar disease, non-tumor histology, liver resection margin status (R0 and R1; Vas R1 and Par R1), and necrosis in the resection margin based on the pathologic report. Additionally, the tumor burden score (TBS) was calculated using the formula: (TBS)^2^ = (maximum tumor diameter in cm)^2^ + (number of lesions)^2^. According to the TBS, all the patients were classified into zones 1, 2, and 3, which were defined as ≤ 25%, > 25% and < 90%, and ≥ 90% in zones 1, 2, and 3, respectively, based on the smallest order [30]. The treatment data received by each patient, such as preoperative and adjuvant chemotherapy and the extent of liver resection, were included. For preoperative chemotherapy, oxaliplatin- or irinotecan-based therapy was administered, and monoclonal antibody-based therapy with bevacizumab or cetuximab was administered as targeted therapy.

### Liver resection

In our institution, patients with synchronous CRLM have been treated by a multidisciplinary team including surgeons, oncologists, radiation oncologists, and radiologists. Particularly, the surgical indication for liver lesions is mainly decided by the liver surgeons. Since the addition of enthusiastic liver surgeons to multidisciplinary team in 2008, the indication of simultaneous resection of synchronous CRLM has expanded to include multiple bilobar metastastic lesions and under minimally invasive method when possible. The type of surgical approach (open, laparoscopic, or robotic) was based on the tumor features and location of both primary CRC and liver metastatic lesions. A laparoscopic approach was preferred for patients with tumor size < 5 cm, no major vascular or other organ invasions, and a favorable tumor location (segments 2, 3, 4, 5, and 6); the liver resection criteria were expanded, including even large tumors, bilobar involvement, and multiple as well as unfavorable tumor locations (segments 1, 7, and 8). Patients with unfavorable tumor locations (segments 1, 7, 8) can be selected in the robotic approach. However, patients who refuse the robotic approach would be offered simultaneous laparoscopic colorectal and open liver resection or open colorectal and liver resection, as appropriate [31, 32]. Of the 338 patients, 285 (77.4%), 39 (10.6%), and 44 (12.0%) patients underwent conventional open, laparoscopy-assisted, and minimally invasive liver resection, respectively.

The extent of surgery, such as minor and major liver resection (minor included two or less segments and major included three or more segments [33, 34]), is based on the tumor size, features, and location. Intraoperative ultrasound was routinely used. During liver resection, all tumors were attempted to be removed leaving sufficient future remnant volume regardless of the type of resection. For bilobar multiple liver metastases, a thorough assessment of tumor locations and their proximity to the portal pedicles and major hepatic veins was done before the operation and double-checked using intraoperative US. The remaining segments were selected based on the possibility of preserving vascular inflow, outflow, and biliary drainage, as well as sufficient remnant volume. Intraoperative radiofrequency ablation (RFA) therapy and Vas R1 were considered for resectability for these patients. In patients with normal liver function, remnant future liver volume was at least 30% of the total liver volume and over 35–40% in patients with chemotherapy-induced sinusoidal injury or steatohepatitis. Parenchymal transection was mainly performed using a Cavitron Ultrasonic Surgical Aspirator (CUSA; Valleylab, Boulder, CO, USA) in the open and laparoscopic approaches. The surgical techniques of robotic simultaneous resection for CRLM at our center have been described in detail in a previous study [32]. During robotic liver resection, the liver parenchyma was transected using a harmonic scalpel and Maryland bipolar forceps. Low central venous pressure was maintained during liver resection through balanced fluid management. The Pringle maneuver was selectively applied.

In our institute, patients with CRLM usually receive postoperative adjuvant chemotherapy. For those with a single CRLM, upfront simultaneous resection followed by adjuvant chemotherapy is recommended. However, for patients with multiple CRLM (two or more), perioperative chemotherapy is the standard approach based on the Korean multicenter study [35]. Patients who undergo preoperative chemotherapy receive routine check-ups to monitor their general condition and nutritional status at each clinic visit. If necessary, a multidisciplinary approach for nutritional support was applied. To prevent chemotherapy-related surgical complications, liver resection was recommended at least 4 weeks after the last chemotherapy administration and 5 weeks for patients who had bevacizumab in their regimen.

### Postoperative follow-up

Postoperative follow-up consisted of clinical examination and CEA measurements every 3 months. Imaging studies (abdomen CT, chest CT) were performed at 3, 6, 9, and 12 months in the first year, every 6 months in the second year, and once a year in the third year after the operation. If the disease recurred, the decision to initiate chemotherapy was determined by the multidisciplinary team.

### Outcomes

OS was defined as the interval in months between the resection of CRLM and death or the date of follow-up. Recurrence-free survival (RFS) was defined as the interval between the resection of CRLM and recurrence, death without recurrence, or date of last follow-up without recurrence. Recurrence was classified as intrahepatic, extrahepatic, and combined recurrence. Marginal recurrence was observed near the resection margins.

Complications were graded by the Clavien-Dindo classification system [36]. To assess any liver-related complications, pleural effusion, bile leakage, ascites, biliary stricture, postoperative bleeding, perihepatic fluid collection, and liver failure were also recorded [37].

### Statistical analysis

The results are presented as mean ± standard deviation or as numbers and percentages. Comparisons between continuous variables were determined by Student’s *t*-tests as a parametric test and Mann–Whitney test as a non-parametric test. Categorical variables were compared using *χ*^2^ or Fisher’s exact test, as appropriate. To reduce the selection bias between the two R0 and R1 resection groups, 2:1 propensity score matching (PSM) was performed to create a comparable control cohort, including preoperative clinical and tumor-related characteristics (age, sex, initial CEA, Colon R1 resection, primary CRC location, primary CRC T stage and N stage, number and size of liver tumor, bilobar disease, intraoperative transfusion, and preoperative chemotherapy). A survival analysis was performed using the Kaplan–Meier method, and differences in survival were assessed using the log-rank test. Univariate analysis was performed using the Cox proportional hazards regression analysis. For multivariate analysis, factors were selected based on the clinical relevance and statistical significance in the univariate analysis (*P* < 0.1). A value of *P* < 0.05 was defined as statistically significant. All the statistical analyses were performed using SPSS for Windows version 26.0 (IBM Corp., Armonk, NY, USA).

## Results

### Patient characteristics

Of the 371 patients, except for three patients due to who underwent R2 resection, 304 and 64 patients who underwent R0 and R1 resection, respectively, were included in the study. Four patients had resectable lung metastasis at the time of diagnosis, but all underwent lung resection separately. Specifically, one patient underwent lung resection first, while three patients underwent lung resection later.

The clinicopathological characteristics are summarized in Table 1. Primary CRC characteristics were similar between the R0 and R1 groups. The R1 group had more patients with ≥ 4 liver lesions than the R0 group (27.3 vs. 50.0%, *P* < 0.001). The mean TBS was statistically higher in the R1 than in the R0 (4.4 vs. 5.8, *P* = 0.003) group, and the proportion of patients in zone 3 was also high (8.2 vs. 15.6%, *P* = 0.015). Bilobar disease was significantly more common in the R1 group compared with that in the R0 (38.8 vs. 67.2%, *P* < 0.001) group. Considering the perioperative treatments, the R1 group had more patients who received preoperative chemotherapy than the R0 group; however, the difference was not significant (48.4 vs. 59.4%, *P* = 0.109). Preoperative targeted therapy was more frequently administered to patients in the R1 group for both bevacizumab and cetuximab (bevacizumab, 14.1 vs. 21.9%; cetuximab, 11.2 vs. 18.8%, *P* = 0.046). Adjuvant chemotherapy was administered in approximately 90% of the patients. The rate of major complication (grade III or higher) was 12.2% with no significant difference between R0 and R1 resection (12.5 vs. 10.9%; *P* = 0.729). The liver-related major complication rate was 6.3%, also showing no significant difference (6.6 vs. 4.7%; *P* = 0.778).

**Table 1:** Demographic and clinicopathologic characteristics of all patients with CRLM undergoing R0 and R1 resection before and after 2:1 propensity score matching (*n* = 368)

Variables	Before matching			After 2:1 matching		
	R0 (*n* = 304)	R1 (*n* = 64)	*P* value	R0 (*n* = 119)	R1 (*n* = 62)	*P* value
	Mean ± SD; frequency (%); median (range)					
Age (years, mean)	58.8 ± 11.2	56.7 ± 9.9	0.168	58.0 ± 10.2	57.0 ± 9.8	0.547
Sex (M/F)	199 (65.5)/105 (34.5)	43 (67.2)/21 (32.8)	0.791	82 (68.9)/37 (31.1)	43 (69.4)/19 (30.6)	0.951
BMI (kg/m^2^)	23.2 ± 2.9	23.2 ± 3.1	0.943	23.2 ± 3.1	23.1 ± 3.1	0.126
**Primary CRC**						
Primary CRC location (Rt colon/Lt colon/rectum)	55 (18.1)/123 (78.8)/126 (41.4)	7 (10.9)/33 (51.6)/24 (37.5)	0.188	24 (20.2)/54 (45.4) /41 (34.5)	7 (11.3)/31 (17.1) /24 (38.7)	0.322
Primary CRC grade (WD/MD/PD)	15 (5.0)/275 (91.1)/12(4.0)	3 (4.7)/58 (90.6)/3(4.7)	0.933	7 (5.9)/109 (91.6)/3 (2.5)	3 (4.8)/56 (90.3)/3 (4.8)	0.644
Primary CRC T stage^b^ (T1–2/T3–4)	20 (6.8) /273 (93.2)	7 (11.5)/54 (88.5)	0.285	7 (6.1)/108 (93.9)	7 (11.9)/52 (88.1)	0.239
Primary CRC N stage (N0/N +)	87 (28.6)/21 7 (71.4)	20 (31.3)/44 (68.8)	0.673	37 (31.1)/82 (68.9)	18 (29.0)/44 (71.0)	0.775
KRAS mutation (wild type/mutant)^c^	116 (65.2)/62 (34.8)	27 (65.9)/14 (34.1)	0.934	49 (67.1)/24 (32.9)	26 (65.0)/14 (35.0)	0.819
CRC R1 status	17 (5.6)	3 (4.3)	1.000	6 (5.0)	3 (4.8)	1.000
**Hepatic lesion**						
Size (cm, mean)	2.5 ± 1.9	3.0 ± 2.3	0.058	2.6 ± 2.1	2.8 ± 1.9	0.513
Tumor size ≥ 5 cm	29 (9.5)	8 (12.5)	0.474	14 (11.8)	6 (9.7)	0.671
No. liver metastasis (≤ 3/ ≥ 4)	221 (72.7)/83 (27.3)	32 (50.0)/ 32 (50.0)	< 0.001	63 (52.9)/56 (47.1)	32 (51.6)/30 (48.4)	0.865
Initial CEA (≤ 50/ > 50)	221 (73.4)/80 (26.6)	42 (65.6)/22 (34.4)	0.207	79 (66.4)/40 (33.6)	42 (67.7)/20 (32.3)	0.854
TBS (mean)	4.4 ± 3.4	5.8 ± 3.4	0.003	5.8 ± 4.2	5.6 ± 3.4	0.790
TBS zone (1/2/3)	90 (29.6)/189 (62.2)/25 (8.2)	9 (14.1)/45 (70.3)/10 (15.6)	0.015	22 (18.5)/79 (66.4)/18 (15.1)	9 (4.5)/44 (71.0)/9 (14.5)	0.774
Bilobar disease	118 (38.8)	43 (67.2)	< 0.001	76 (63.9)	41 (66.1)	0.762
Necrosis ≥ 20%^a^ (*n* = 308)	141 (55.5)	28 (51.9)	0.624	59 (57.3)	27 (51.9)	0.526
Non-tumor histology						
Normal	164 (53.9)	35 (54.7)	0.840	57 (47.9)	35 (56.5)	0.722
Steatosis	98 (32.2)	19 (29.7)		38 (31.9)	17 (27.4)	
Steatohepatitis	14 (4.6)	2 (3.1)		7 (5.9)	2 (3.2)	
Sinusoidal obstruction syndrome	28 (9.2)	8 (12.5)		17 (14.3)	8 (12.9)	
Minor/major liver resection	193 (63.5)/111 (36.5)	44 (68.8)/20 (31.3)	0.424	74 (62.2)/45 (37.8)	43 (69.4)/19 (30.6)	0.338
Intraoperative RFA	49 (16.1)	13 (20.3)	0.415	32 (26.9)	12 (19.4)	0.262
Intraoperative transfusion	57 (18.8)	22 (34.4)	0.006	34 (28.6)	20 (32.3)	0.607
Preoperative chemotherapy	147 (48.4)	38 (59.4)	0.109	73 (61.3)	36 (58.1)	0.669
Preoperative target therapy (bevacizumab/cetuximab)	43 (14.1) /34 (11.2)	14 (21.9)/12 (18.8)	0.046	22 (18.5)/16 (13.4)	14 (22.6)/11 (17.7)	0.525
Adjuvant chemotherapy	270 (89.1)	60 (93.8)	0.263	103 (86.6)	58 (93.5)	0.154

*CRC* colorectal cancer, *TBS* tumor burden score, *BMI* body mass index, *SD* standard deviation, *M* male, *F* female, *CEA* carcinoembryonic antigen, *Rt* right, *Lt* left, *RFA* radiofrequency ablation, *WD* well differentiation, *MD* moderate differentiation, *PD* poor differentiation

^a^Necrosis in the resection margin, 60 patients were not reported

^b^T0 stage was excluded

^c^Among all patients, 219 patients were performed KRAS mutation testing

After PSM, a new cohort compared 119 patients with R0 resection and 62 patients with R1 resection. In the PSM cohort, clinicopathologic characteristics were similar between R0 and R1 resection.

**Fig. 1:**
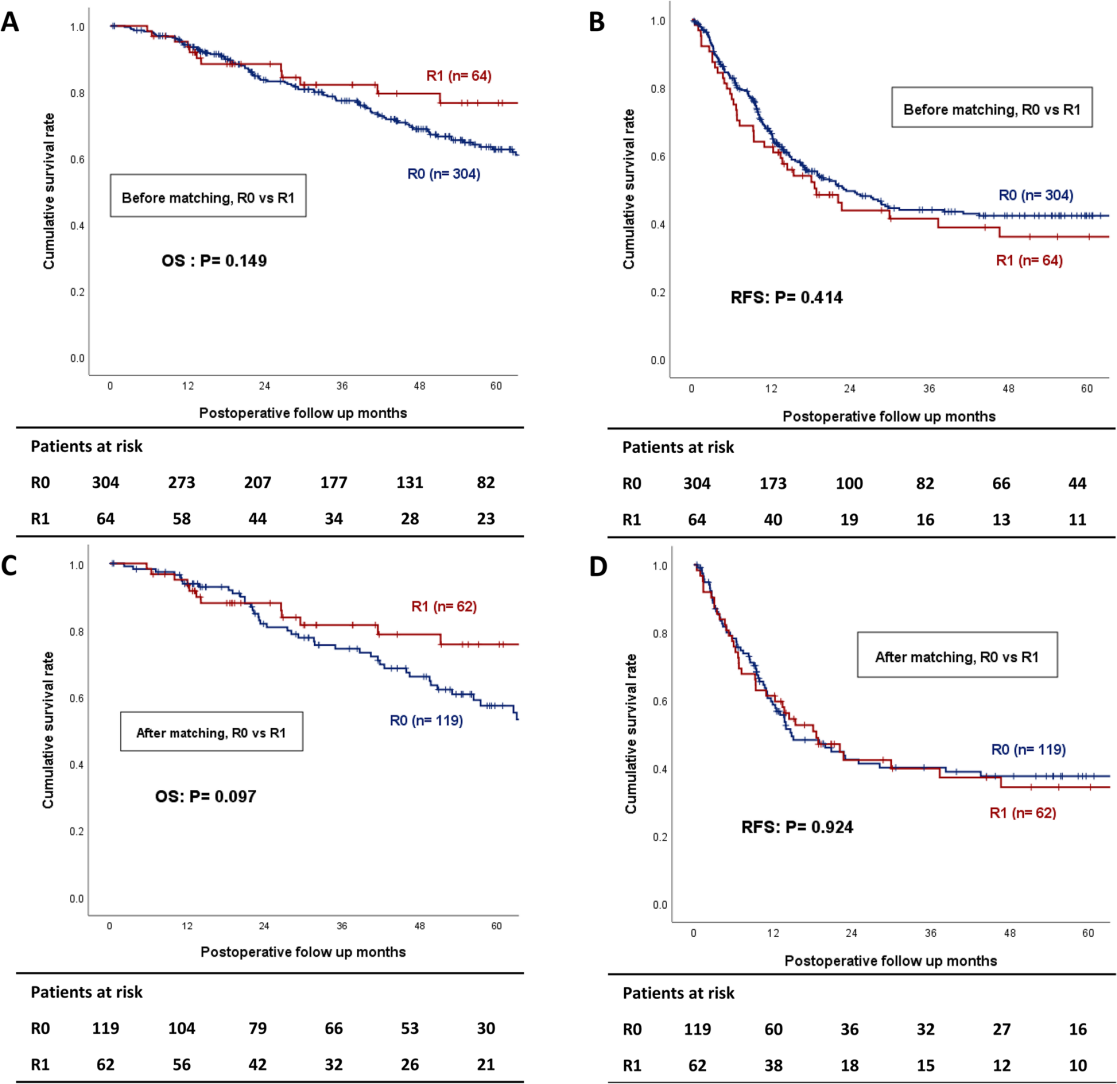
Overall survival and intrahepatic recurrence-free survival Kaplan–Meier curve depending on Resection margin status, before (**A**, **B**) and after (**C**, **D**) propensity score matching (R0 vs. R1; 5-year OS: 62.7 vs. 76.7%, *P* = 0.149; 5-year RFS: 42.2 vs. 36.0%, *P* = 0.414; after matching, 5-year OS: 57.3 vs. 75.7%, *P* = 0.097; 5-year RFS: 37.5 vs. 34.3%, *P* = 0.924)

In the total cohort, the OS and intrahepatic RFS were similar between the R0 and R1 groups (5-year OS: 62.7 vs. 76.7%, *P* = 0.149; 5-year RFS: 42.2 vs. 36.0%, *P* = 0.414) (Fig. 1A, B). Even after matching, the resection margin did not have a significant impact on long-term outcomes (5-year OS: 57.3 vs. 75.7%, *P* = 0.097; 5-year RFS: 37.5 vs. 34.3%, *P* = 0.924) (Fig. 1C, D).

### Subgroup analysis: vascular R1 versus parenchymal R1 resection

The Vas R1 group had a significantly larger tumor size than Par R1 (3.0 vs. 1.9 cm, *P* = 0.047). Bilobar disease was more common in the Vas R1 group compared with that in the Par R1 group (78.9 vs. 62.2%, *P* = 0.193); however, the difference was not significant. In terms of treatment, the rate of major liver resection was higher in Vas R1 than in Par R1 (47.4 vs. 24.4%, *P* = 0.071), and adjuvant chemotherapy was performed more than 90% in both groups with no difference (94.7 vs. 93.3%, *P* = 1.000). The median follow-up duration was 71.1 (29.5–87.4) months and 31.9 (13.7–56.3) months in Vas R1 and Par R1, respectively. The OS was comparable in both groups (5-year OS: 88.1 vs. 70.4%; *P* = 0.285); however, the RFS was superior in Vas R1 than in Par R1 (5-year RFS:51.6 vs. 29.1%; *P* = 0.025) (Additional file 1: Fig. S1).

### Prognostic effects of liver resection margin according to whether or not preoperative chemotherapy was administered

**Fig. 2:**
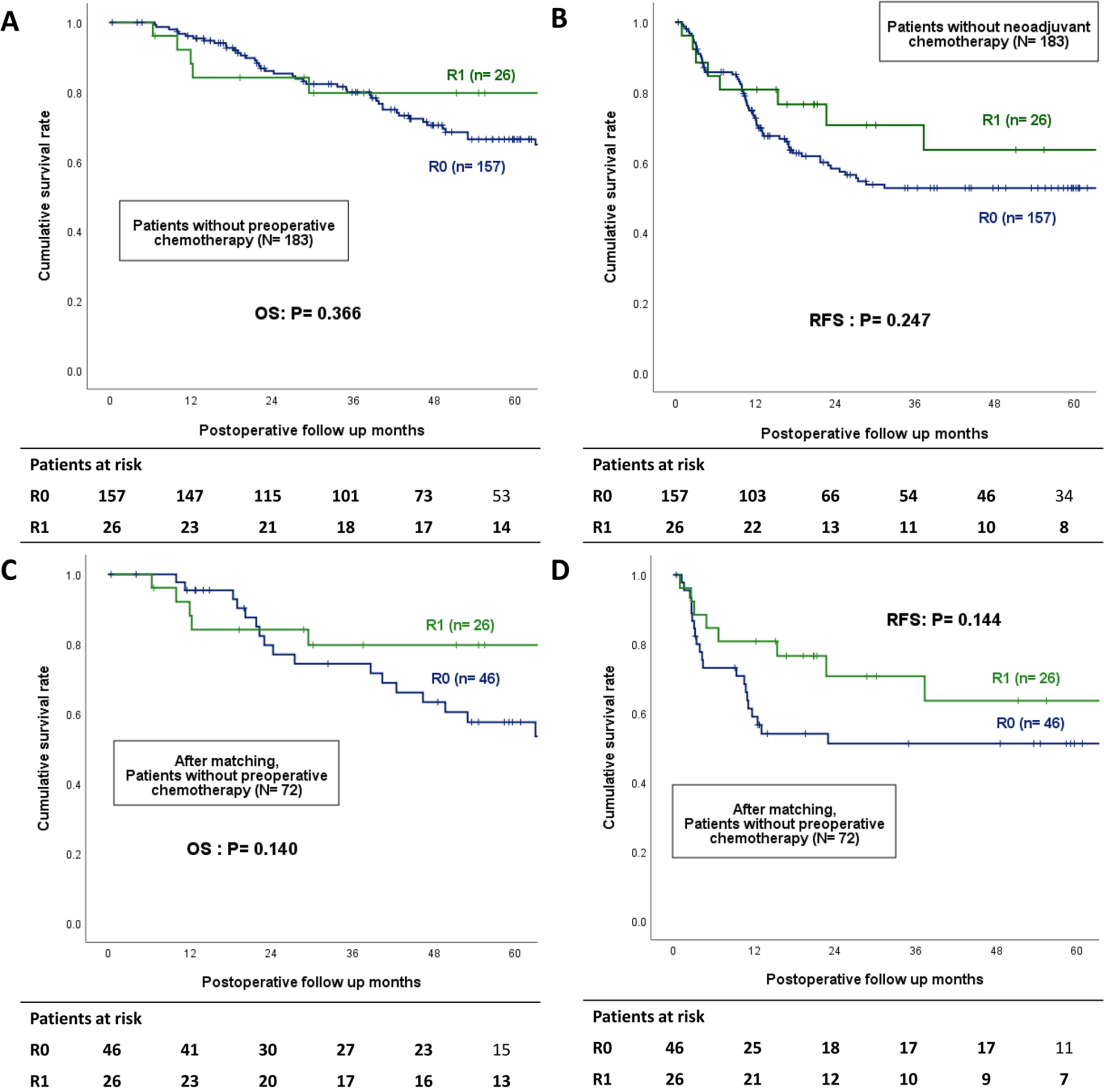
Overall survival and intrahepatic recurrence-free survival Kaplan–Meier curve in patients not receiving neoadjuvant chemotherapy before (**A**, **B**) and after (**C**, **D**) propensity score matching

**Fig. 3:**
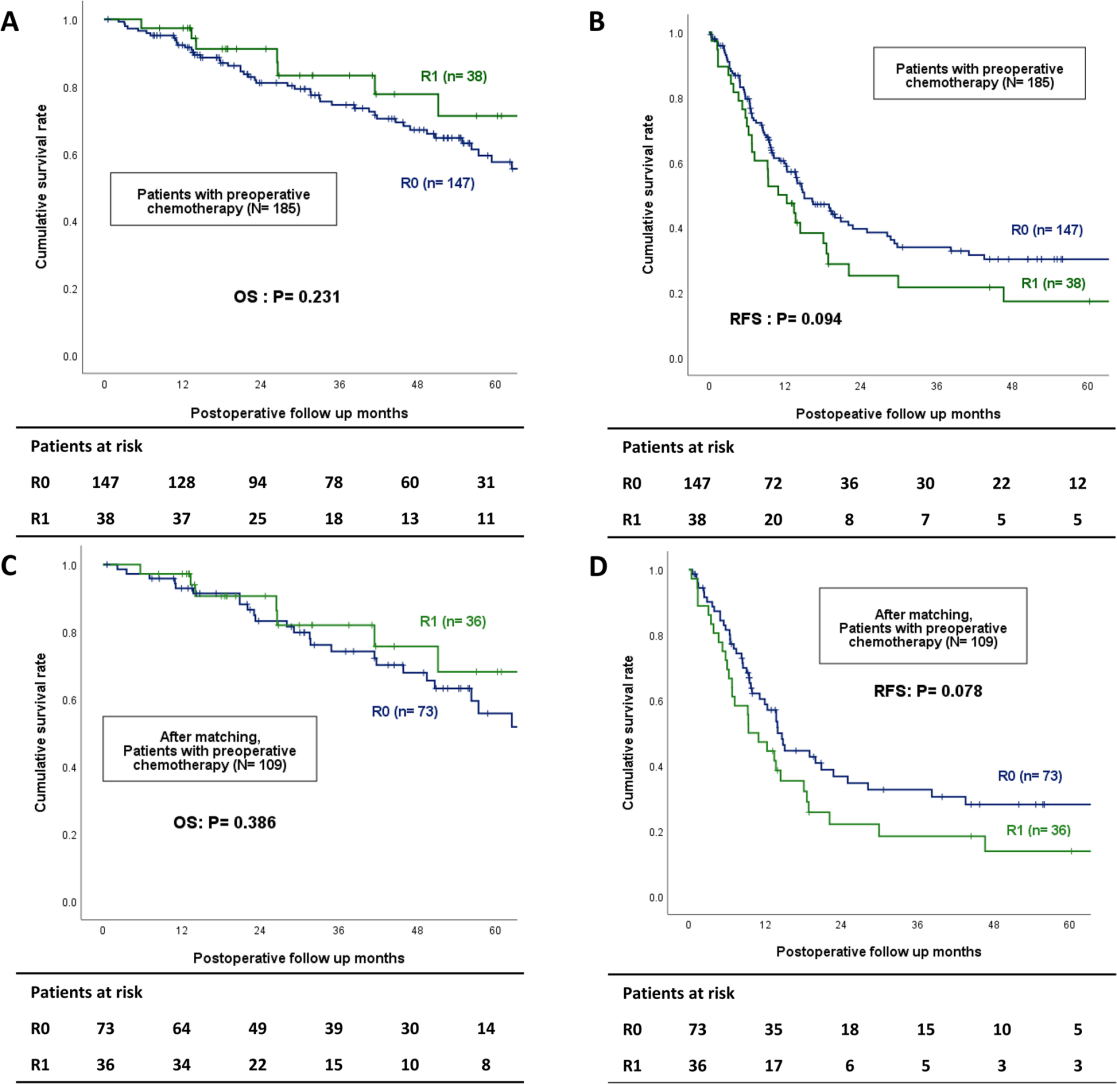
Overall survival and intrahepatic recurrence-free survival Kaplan–Meier curve in patients receiving neoadjuvant chemotherapy before (**A**, **B**) and after (**C**, **D**) propensity score matching

Among the 368 patients, liver recurrence occurred in 188 (51.1%) patients, and the incidence of liver recurrence was similar in both the R0 and R1 groups (49.7 vs. 57.8%, *P* = 0.236). However, marginal recurrence was more common in the R1 group (16.1 vs. 26.6%, *P* = 0.048) (Table 2). Considering the use of preoperative chemotherapy, the OS and intrahepatic RFS were analyzed in both groups. The median follow-up was 42.0 months. For the 183 patients who did not receive preoperative chemotherapy, no difference was observed in the OS (5-year OS, 66.3 vs. 79.7%, *P* = 0.366) and intrahepatic RFS in either group (5-year RFS, 52.7 vs. 63.6%, *P* = 0.247) (Fig. 2A, B). For the 185 (50.3%) patients who received preoperative chemotherapy, the OS and intrahepatic RFS were similar in both groups (5-year OS 57.5 vs. 71.2%, *P* = 0.231; 5-year RFS 30.2 vs. 17.2%, *P* = 0.094) (Fig. 3A, B). Especially, in the case of patients who received preoperative chemotherapy, early RFS was worse than in those who did not receive preoperative chemotherapy: 58.2 vs. 39.5% in R0 (*P* = 0.001) and 70.6 vs. 25.1% in R1 (*P* = 0.001) in 2 years. Depending on whether preoperative chemotherapy was administered, the liver-related major complication rate was 8 (4.4%) for those who did not receive preoperative chemotherapy and 15 (8.1%) for those who did (*P* = 0.139). Additionally, adjuvant chemotherapy for CRLM was beneficial for both OS and RFS (5-year OS: 45.4 vs. 66.7%: *P* = 0.011; 5-year RFS: 27.0 vs. 42.9%: *P* = 0.027; Additional file 2: Fig. S2).

**Table 2:** The incidence of liver recurrence in R0 and R1 liver resection before and after 2:1 propensity score matching

	**Total (*n* = 368)**	**Before matching**			**After matching**		
		**R0 (*n* = 304)**	**R1 (*n* = 64)**	***P* value**	**R0 (*n* = 119)**	**R1 (*n* = 62)**	***P* value**
Recurrence	257 (69.8)	211 (69.4)	46 (71.9)	0.696	85 (71.4)	45 (72.6)	0.870
Liver recurrence	188 (51.1)	151 (49.7)	37 (57.8)	0.236	66 (55.5)	37 (59.7)	0.587
Recurrence site							
Intrahepatic	121 (32.9)	95 (31.3)	26 (40.6)	0.380	43 (36.1)	26 (41.9)	0.736
Extrahepatic	69 (18.8)	61 (20.1)	8 (12.5)		20 (16.8)	7 (11.3)	
Intrahepatic + extrahepatic	67 (18.2)	56 (18.4)	11 (17.2)		23 (19.3)	11 (17.7)	
Marginal recurrence	66 (17.9)	49 (16.1)	17 (26.6)	0.048*	25 (21.0)	17 (27.4)	0.332

*P- value less than 0.05 was considered statistically significant

In the PSM cohort, liver recurrence (55.5vs. 59.7%, *P* = 0.587) and marginal recurrence (21.0 vs. 27.4%, *P* = 0.332) were similar between R0 and R1 groups (Table 2). Similar with before matching, for the 72 patients who did not receive preoperative chemotherapy in the PSM cohort, the OS (5-year OS, 57.6 vs. 79.7%, *P* = 0.140) and intrahepatic RFS (51.2 vs. 63.6%, *P* = 0.144) were similar between the R0 and R1 groups (Fig. 2C, D). For 109 patients who received preoperative chemotherapy, the OS and intrahepatic RFS were similar between both groups (5-year OS: 55.7 vs. 68.1%, *P* = 0.386; 5-year RFS: 28.1 vs. 13.8%, *P* = 0.078; Fig. 3C, D).

### Prognostic factor

In the multivariate Cox regression analysis for OS, independent negative prognostic factors included (1) poorly differentiated CRC [hazard ratio (HR): 5.483; 95% confidence interval (CI): 2.670–11.262; *P* < 0.001], (2) N-positive stage of CRC (HR: 3.136; 95% CI: 1.748–5.626; *P* < 0.001), (3) liver lesions ≥ 4 (HR: 1.851; 95% CI: 1.230–2.786; *P* = 0.003), and (4) tumor size ≥ 5 cm (HR: 2.713; 95% CI: 1.670–4.408; *P* < 0.001) (Table 3). Similarly, in the multivariate Cox regression analysis for intrahepatic RFS, several factors were associated with poor prognosis, including (1) N-positive stage of CRC (HR: 2.201; 95% CI: 1.540–3.146; *P* < 0.001), (2) liver lesions ≥ 4 (HR: 1.542; 95% CI: 1.069–2.223; *P* = 0.020), (3) TBS zone 3 (HR: 2.289; 95% CI: 1.232–4.253; *P* = 0.009), and (4) preoperative chemotherapy (HR: 1.526; 95% CI: 1.103–2.112; *P* = 0.011) (Table 4).

**Table 3:** Univariable and multivariable Cox proportional hazards regression analysis for overall survival in all the patients

Variables	Univariate		Multivariate	
	**HR**	***P***	**HR**	***P***
Age (years) (≤ 60/ > 60)	0.959 (0.652–1.409)	0.830		
Sex (F/M)	0.890 (0.600–1.319)	0.561		
BMI (kg/m^2^) (≤ 25/ > 25)	1.135 (0.737–1.747)	0.566		
Initial CEA (≤ 50/ > 50)	1.456 (0.980–2.164)	0.063*		
**Primary CRC**				
CRC grade (WD-MD/PD)	4.453 (2.306–8.597)	< 0.001*	5.483 (2.670–11.262)	< 0.001
CRC T stage (T1–2/T3–4)	3.027 (0.960–9.549)	0.059*		
CRC N stage (N0/N +)	2.871 (1.663–4.958)	< 0.001*	3.136 (1.748–5.626)	< 0.001
CRC resection status (R0/R1)	1.987 (1.003–3.936)	0.049*		
CRC location				
Right colon	1 (reference)			
Left colon	0.642 (0.378–1.089)	0.100		
Rectum	0.887 (0.529–1.487)	0.650		
**Liver metastatic tumor**				
Liver resection margin status (R0/R1)	0.655 (0.366–1.170)	0.152		
Number of liver tumor (≤ 3/ ≥ 4)	1.759 (1.195–2.591)	0.004*	1.851 (1.230–2.786)	0.003
Liver tumor size (cm) (< 5/ ≥ 5)	2.437 (1.525–3.896)	< 0.001*	2.713 (1.670–4.408)	< 0.001
Tumor burden score				
Zone 1	1 (reference)	< 0.001*		
Zone 2	1.764 (1.051–2.961)	0.032		
Zone 3	4.608 (2.412–8.804)	< 0.001		
Extent of liver resection				
Minor/major	1.282 (0.871–1.888)	0.208		
Bilobar disease	1.430 (0.979–2.090)	0.064*		
Necrosis (*n* = 308) (≤ 20/ > 20%)	0.902 (0.599–1.358)	0.621		
Non-tumor histology				
Normal		0.246		
Steatosis	0.729 (0.464–1.144)	0.169		
Steatohepatitis	0.881 (0.276–2.810)	0.830		
Sinusoidal obstruction syndrome	1.398 (0.783–2.498)	0.257		
Intraoperative transfusion (N/Y)	1.440 (0.931–2.228)	0.101		
Preoperative chemotherapy (N/Y)	1.167 (0.798–1.707)	0.425		
Preoperative target therapy (N/Y)	1.043 (0.665–1.636)	0.854		
Adjuvant chemotherapy (N/Y)	0.498 (0.288–0.860)	0.012*	0.465 (0.259–0.837)	0.011

*M* male, *F* female, *CEA* carcinoembryonic antigen, *HR* hazard ratio, *CRC* colorectal cancer, *TBS* Tumor Burden Score, *BMI* body mass index, *WD* well differentiation, *MD* moderate differentiation, *PD* poor differentiation, *N* no, *Y* yes

^*^Statistically significant results from the Cox proportional regression analysis. Variables with *P* < 0.1 in the univariate analysis were applied to a multivariate analysis

**Table 4:** Univariable and multivariable Cox proportional hazards regression analysis for intrahepatic recurrence-free survival in all the patients

Variables	Univariate		Multivariate	
	**HR**	***P***	**HR**	***P***
Age (years) (≤ 60/ > 60)	0.759 (0.565–1.019)	0.067*		
Sex (F/M)	1.163 (0.855–1.580)	0.336		
BMI (kg/m^2^) (≤ 25/ > 25)	1.472 (1.080–2.008)	0.014*		
**Primary CRC**				
CRC grade (WD-MD/PD)	1.159 (0.570–2.353)	0.684		
CRC T stage (T1–2/T3–4)	1.659 (0.877–3.140)	0.120		
CRC N stage (N0/N +)	2.001 (1.414–2.833)	< 0.001*	2.201 (1.540–3.146)	< 0.001
CRC resection status (R0/R1)	1.535 (0.873–2.698)	0.136		
CRC location				
Right colon	1 (reference)			
Left colon	0.669 (0.445–1.007)	0.054		
Rectum	0.864 (0.575–1.299)	0.483		
**Liver metastatic tumor**				
Liver resection margin status (R0/R1)	1.180 (0.824–1.691)	0.367		
Liver tumor size(cm) (< 5/ ≥ 5)	1.289 (0.810–2.049)	0.284		
Number of liver tumor (≤ 3/ ≥ 4)	2.425 (1.815–3.242)	< 0.001*	1.542 (1.069–2.223)	0.020
Tumor burden score				
Zone 1	1 (reference)	< 0.001*		
Zone 2	1.663 (1.147–2.410)	0.007*		
Zone 3	3.966 (2.412–6.523)	< 0.001*	2.289 (1.232–4.253)	0.009
Initial CEA (≤ 50/ > 50)	1.643 (1.209–2.233)	0.002*		
Necrosis (*n* = 308) (≤ 20/ > 20%)	0.857 (0.629–1.168)	0.329		
Extent of liver resection				
Minor/major	1.061 (0.788–1.429)	0.696		
Bilobar disease (N/Y)	2.037 (1.525–2.720)	< 0.001*		
Non-tumor histology				
Normal		0.066*		
Steatosis	1.171 (0.847–1.620)	0.338		
Steatohepatitis	2.093 (1.144–3.829)	0.016		
Sinusoidal obstruction syndrome	1.542 (0.976–2.436)	0.064*		
Intraoperative transfusion (N/Y)	1.033 (0.726–1.469)	0.858		
Preoperative chemotherapy (N/Y)	1.988 (1.482–2.666)	< 0.001*	1.526 (1.103–2.112)	0.011
Preoperative target therapy (N/Y)	1.593 (1.175–2.160)	0.003*		
Adjuvant chemotherapy (N/Y)	0.559 (0.361–0.866)	0.009*	0.488 (0.308–0.771)	0.002

*CRC* colorectal cancer, *TBS* Tumor Burden Score, *BMI* body mass index, *M* male, *F* female, *CEA* carcinoembryonic antigen, *HR* hazard ratio, *WD* well differentiation, *MD* moderate differentiation, *PD* poor differentiation, *N* no, *Y* yes

^*^Statistically significant results from the Cox proportional regression analysis. Variables with *P* < 0.1 in univariate analysis were applied to multivariate analysis

However, adjuvant chemotherapy had a positive impact on the OS and intrahepatic RFS (OS, HR: 0.465, 95% CI: 0.259–0.837, *P* = 0.011; RFS, HR: 0.488, 95% CI: 0.308–0.771, *P* = 0.002) (Tables 3 and 4). Liver resection margin status was not an independent prognostic factor for OS and RFS.

## Discussion

This study reviewed patients who underwent simultaneous colorectal and liver resection for synchronous CRLM combined with perioperative systemic chemotherapy. In this study, the rate of R1 resection was 17.4%, which is consistent with the previously reported range of 10–30% [27, 29]. The results demonstrated that the R1 resection group was associated with aggressive tumor characteristics such as ≥ 4 liver lesions, high TBS, and bilobar disease compared with the R0 group. Similarly, several previous studies reported that aggressive tumor factors such as larger size, multiple lesions, and bilobar disease were high in the R1 resection group [11, 38].

In this cohort of patients who underwent intense follow-up after liver resection with regular clinical visits and imaging studies, intrahepatic lesions recurred in 188 (51.1%) patients, of whom 37 (57.8%) were in the R1 group, which is slightly higher than the previously reported rate of 42.2 to 52.0% [29, 38–40]. For marginal recurrence, 26.6% at R1 resection was similar to that reported in a previous study [29, 38, 40], which demonstrated that R1 was significantly related to the marginal recurrence compared to R0. Similarly, a recently published study by Ausania et al. demonstrated that surgical margin recurrence was more common in the R1 contact group involving the resection margin than in the submillimeter margin group [13]. However, the rate of marginal recurrence was similar in both groups after propensity score matching. Therefore, the tumor characteristics of the R1 resection group rather than resection margin status may be related to marginal recurrence.

Among the R1 resection group in our cohort, parR1 had comparable OS but inferior RFS than those with vasR1. According to a previous study by Luca et al., Vas R1 showed a similar long-term outcome compared to R0 resection, but par R1 was an independent negative prognostic factor for OS and a risk factor of local recurrence [19]. VasR1 can be predicted through preoperative images and is acceptable in terms of long-term outcomes, while parR1 can be avoided by using intraoperative ultrasound and should be reduced to minimize the risk of tumor exposure at the resection margin.

According to our data, poor differentiation, N-positive CRC stage, liver lesion number ≥ 4, and size ≥ 5 cm were negative predictive factors for the OS. For the intrahepatic RFS, poor prognostic factors included N-positive CRC stage, liver lesions ≥ 4, TBS zone 3, and preoperative chemotherapy. Postoperative chemotherapy was included as a positive prognostic factor for both the OS and intrahepatic RFS. However, the resection margin status did not have any effect on the OS and intrahepatic RFS. Although the incidence of marginal recurrence was higher in R1, the biological characteristics of the tumor itself could have an impact on its survival rather than R1 resection. Additionally, Mao et al. revealed that the higher the tumor burden, the smaller the effect of R1 resection on the resection margin recurrence [38]. Therefore, although the rate of R1 resection may be high in CRLM patients with high tumor burden, surgical resection should be considered.

Hence, the effect of preoperative chemotherapy associated with resection margin status on survival, or the occurrence of surgical margin recurrence remains controversial. Several authors have stated that R1 resection does not play a significant prognostic role in patients receiving preoperative chemotherapy [11, 40]. This study is a comparative analysis between the R0 and R1 resection groups with or without preoperative chemotherapy. There was no statistically significant difference between R1 and R0 resection for OS and intrahepatic RFS in patients who received chemotherapy before surgery, which is similar to the results of previous studies. Likewise, the OS and RFS had no difference between R0 and R1 resection groups after propensity score matching. This suggests that confirming the exact margin following chemotherapy could be challenging owing to necrosis of the margin. Even if the resection margin is exposed, the necrosed lesion could have few viable tumor cells. Additionally, this result is consistent with the role of bevacizumab in inducing CRLM necrosis according to a previous study [41].

Moreover, since resection was performed using a Cavitron Ultrasonic Surgical Aspirator (CUSA), liver parenchyma aspiration during dissection could result in a narrow margin in the final pathologic report. These factors could have reduced the prognostic power of R1 resection. In contrast to previous studies, the OS and intrahepatic RFS were similar in the R0 and R1 groups in patients who underwent surgery without chemotherapy [11, 40]. The patients who did not previously receive preoperative chemotherapy showed better intrahepatic RFS in the early period (Figs. 2 and 3). This could be because patients undergoing chemotherapy before surgery have a more advanced disease. Adjuvant chemotherapy as another perioperative treatment strategy was demonstrated to be a positive prognostic factor in this study, with 330 (89.9%) patients receiving chemotherapy after surgery, 89.1% and 93.8% in R0 and R1, respectively. In the current study, more patients received postoperative chemotherapy than those in previous studies [11, 29, 42–44]. Nishioka et al. reported that 5-year OS and RFS were 77.9% and 43.7%, respectively, in synchronous CRLM patients who received adjuvant chemotherapy, superior to 44.5% and 15.2% in those who did not receive. This was consistent with the result of our study, but adjuvant chemotherapy was administrated in 60.3% of patients [45]. Hosokawa et al. demonstrated that effective postoperative chemotherapy could improve survival even in the R1 resection group [46]. Even in patients not receiving preoperative chemotherapy, postoperative chemotherapy improved the survival rate, thereby resulting in no difference in the survival between the R0 and R1 groups.

The present study has several limitations. First, this was a retrospective study. Second, the study was conducted on a relatively small number of patients who underwent surgery for a wide period since 2006. Like the paradigm shift of the resectability of CRLM, more aggressive liver resections for bilobar multiple lesions were performed recently [47, 48]. Third, the nearest resection margin was used for the analysis of multiple CRLMs; thus, the tumor biologic characteristics corresponding to the resection margin may not be reflected in the study. This point should be compensated for in further studies by comparing the imaging and pathological reports to identify the corresponding tumor and reflect its characteristics. Lastly, since only whether chemotherapy was performed was included, the survival analysis would be more accurate if information concerning the number of cycles, regimen, and response to chemotherapy was included.

## Conclusions

R1 resection was associated with aggressive tumor characteristics; however, it had no effect on the OS and intrahepatic RFS with or without preoperative chemotherapy. Tumor biologic characteristics, such as tumor number, TBS, and bilobar disease, are independent prognostic factors for long-term survival; however, the resection margin status was not. Postoperative chemotherapy is beneficial for the long-term prognosis of CRLM. Therefore, aggressive surgical resection should be considered in patients with advanced CRLM, who are expected to undergo R1 resection in this multidisciplinary approach era.

## Supplementary Information

**Additional file 1: Supplementary figure S1.**

**Additional file 2: Supplementary figure S2.**

**Additional file 3: Table S1.** Demographic, clinicopathologic characteristics and recurrence pattern of parenchymal R1 versus vascular R1 resection patients with CRLM before and after propensity score matching (*n* = 64).

## Data Availability

The datasets used during the current study are available from the corresponding author upon reasonable request.

## Abbreviations

CRLM: Colorectal liver metastasis
OS: Overall survival
RFS: Recurrence-free survival
CRC: Colorectal cancer
CEA: Carcinoembryonic antigen
TBS: Tumor burden score
CT: Computed tomography
CI: Confidence interval
HR: Hazard ratio
PSH: Parenchymal sparing hepatectomy
CUSA: Cavitron Ultrasonic Surgical Aspirator
PSM: Propensity score matching
Vas R1: Vascular R1
Par R1: Parenchymal R1
