# Supplementary material for: Prognostic impact of R1 resection margin in synchronous and simultaneous colorectal liver metastasis resection: a retrospective cohort study
Source: World J Surg Oncol. 2023 Jun 7;21:169. doi: 10.1186/s12957-023-03042-5 (PMC10245648; doi:10.1186/s12957-023-03042-5)
Supplement: Supplementary file 3 — Additional file 3: Table S1. Demographic, clinicopathologic characteristics and recurrence pattern of parenchymal R1 versus vascular R1 resection patients with CRLM before and after propensity score matching (n = 64). [file 12957_2023_3042_MOESM3_ESM.docx]

**Supplementary table 1**. Demographic, clinicopathologic characteristics and recurrence pattern of parenchymal R1 versus vascular R1 resection patients with CRLM before and after propensity score matching (*n*= 64)

| Variables | Par R1(*n*= 45) | Vas R1(*n*=19) | *P* value |
| --- | --- | --- | --- |
|  | Mean ± SD; Frequency (%); Median (Range) | |  |
| Age (Years, mean) | 57.9±8.5 | 54.0±12.3 | 0.154 |
| Sex (M/F) | 33(73.3)/ 12(26.7) | 10(52.6)/ 9(47.4) | 0.107 |
| BMI (kg/m^2^) | 22.8 (20.8-25.9) | 22.9 (20.3-25.4) | 0.654 |
| **Primary CRC** |  |  |  |
| Primary CRC location (Colon/ Rectum) | 25 (55.6)/ 20(44.4) | 15 (78.9)/ 4(21.1) | 0.096 |
| Primary CRC grade  (WD/ MD/ PD) | 1(2.2)/ 41(91.1)/ 3(6.7) | 2(10.5)/ 17(89.5)/ 0(0) | 0.213 |
| Primary CRC T stage^‡^ (T1-2/T3-4) | 4(6.8)/ 41 (93.2) | 6(23.5) /13 (76.5) | 0.087 |
| Primary CRC N stage (N0/N+) | 13(28.9)/ 32(71.1) | 7(36.8)/ 12(63.2) | 0.531 |
| CRC R1 status | 3 (6.8) | 0 (0) | 0.547 |
| **Hepatic lesion** |  |  |  |
| Tumor size (cm, mean) | 1.9 (1.5-3.8) | 3.0 (2.0-4.2) | 0.047 |
| Tumor size ≥ 5 cm | 5 (11.1) | 3 (15.8) | 0.685 |
| No. liver metastasis (≤ 3/ ≥ 4) | 23(51.1) /22(48.9) | 9(47.4) /10(52.6) | 0.784 |
| Initial CEA (≤ 50/> 50) | 17 (37.8) | 5 (26.3) | 0.378 |
| TBS (mean) | 5.8±3.6 | 6.0±3.6 | 0.823 |
| TBS Zone (1/2/3) | 7(15.6)/31(68.9)/7(15.6) | 2(10.5)/14(73.7)/3(15.8) | 0.916 |
| Bilobar disease | 28 (62.2) | 15 (78.9) | 0.193 |
| Necrosis ≥20% ^†^ (*n=* 308) | 18 (47.4) | 10 (62.5) | 0.310 |
| Non-tumor histology |  |  | 0.794 |
| Normal | 24 (53.3) | 11 (57.9) |  |
| Steatosis | 11 (57.9) | 5 (26.3) |  |
| Steatohepatitis | 2 (4.4) | 0 (0) |  |
| Sinusoidal obstruction syndrome | 5 (11.1) | 3 (15.8) |  |
| Minor/Major liver resection | 34 (75.6)/ 11(24.4) | 10 (52.6)/ 9(47.4) | 0.071 |
| Neoadjuvant chemotherapy | 28 (62.2) | 10 (52.6) | 0.475 |
| Preoperative target therapy  (Bevacizumab/ Cetuximab) | 10 (22.2)/ 9 (20.0) | 4 (21.1)/ 3 (15.8) | 0.808 |
| Adjuvant chemotherapy | 42 (93.3) | 18 (94.7) | 1.000 |
| **Recurrence pattern** |  |  |  |
| Recurrence | 33(73.3) | 13(68.4) | 0.690 |
| Liver recurrence | 30(66.7) | 7(36.8) | 0.027 |
| Recurrence site |  |  |  |
| Intrahepatic | 22(48.9) | 4(21.1) | 0.015 |
| Extrahepatic | 2(4.4) | 6(31.6) |  |
| Intrahepatic+ Extrahepatic | 8(17.8) | 3(15.8) |  |
| Marginal recurrence | 14 (31.1) | 3 (15.8) | 0.205 |
| CRC, colorectal cancer; TBS, Tumor burden score; BMI, body mass index; SD, standard deviation; M, male; F, female; CEA, carcinoembryonic antigen  WD, well differentiation; MD, moderate differentiation; PD, poor differentiation  † Necrosis in the resection margin, 60 patients were not reported; ‡ T0 stage was excluded  N/A, not applicable | | | |
